# Increased nuclear import characterizes aberrant nucleocytoplasmic transport in neurons from patients with spinocerebellar ataxia type 7

**DOI:** 10.3389/fnmol.2024.1478110

**Published:** 2024-11-22

**Authors:** Joshua G. Macopson-Jones, Maile Adams, Julien Philippe, Albert R. La Spada

**Affiliations:** ^1^Department of Pathology and Laboratory Medicine, University of California, Irvine, Irvine, CA, United States; ^2^Department of Neurology, Duke University School of Medicine, Durham, NC, United States; ^3^Department of Neurology, University of California, Irvine, Irvine, CA, United States; ^4^Department of Biological Chemistry, University of California, Irvine, Irvine, CA, United States; ^5^Department of Neurobiology and Behavior, University of California, Irvine, Irvine, CA, United States; ^6^UCI Center for Neurotherapeutics, University of California, Irvine, Irvine, CA, United States

**Keywords:** spinocerebellar ataxia (SCA) 7, polyglutamine (polyQ) diseases, nucleocytoplasmic (N/C) transport, mouse model, induced pluripotent stem cells (iPSCs), neuron progenitor cells (NPCs), cortical neurons, fluorescence recovery after photo bleaching (FRAP)

## Abstract

**Introduction:**

Spinocerebellar ataxia type 7 (SCA7) is an inherited neurodegenerative disorder characterized by cerebellar and retinal degeneration. SCA7 is caused by a CAG-polyglutamine repeat expansion in the ataxin-7 gene, which encodes a transcription factor protein that is a core component of the STAGA co-activator complex. As ataxin-7 protein regularly shuttles between the nucleus and the cytosol, we sought to test if polyglutamine-expanded ataxin-7 protein results in nuclear membrane abnormalities or defects in nucleocytoplasmic (N/C) transport.

**Methods:**

We used SCA7 266Q knock-in mice and their wild-type (WT) littermate controls to assess nuclear membrane morphology and N/C transport. Additionally, induced pluripotent stem cells (iPSCs) from SCA7 patients were differentiated into neural progenitor cells (NPCs) and cortical neurons to measure nuclear import and export dynamics. The expression of nucleoporin POM121, a key regulator of N/C transport, was also analyzed in SCA7-derived NPCs.

**Results:**

Our analysis revealed no significant differences in nuclear membrane morphology between SCA7 knock-in mice and WT controls, nor did we observe alterations in N/C transport within neurons from these mice. However, we documented significantly increased nuclear import in both NPCs and cortical neurons derived from SCA7 patient iPSCs. When we examined nuclear export function in SCA7 iPSC-derived cortical neurons, we noted a modest decrease that constituted only a trend. Furthermore, we identified a significant decrease in the expression of full-length POM121 in SCA7 NPCs.

**Discussion:**

Our results reveal evidence for altered N/C transport in SCA7. The reduction in POM121 expression suggests a potential mechanism underlying these transport abnormalities. Importantly, our data suggests the N/C transport defect in SCA7 is distinctly different from other related neurodegenerative disorders.

## 1 Introduction

Spinocerebellar ataxia type 7 (SCA7) is a hereditary autosomal dominant neurodegenerative disorder characterized by cerebellar and retinal degeneration, resulting in ataxia, speech articulation difficulties, eye muscle movement abnormalities, and heightened reflexes indicative of extra-cerebellar involvement (La Spada, 1993). As one of the most unstable CAG repeat disorders, SCA7 accounts for ~4% of all Spinocerebellar Ataxia (SCA) cases in the USA. SCA7 is caused by a CAG-polyglutamine (polyQ) repeat expansion in the ataxin-7 gene (Nardacchione et al., 1999; van de Warrenburg et al., 2001). Unaffected individuals typically have fewer than 36 CAG repeats, while those with ≥37 CAG repeats are affected with SCA7 disease (Johansson et al., 1998). Additionally, patients with >59 repeats generally experience initial onset of vision impairment, followed by juvenile or early adult onset of cerebellar ataxia, sometimes accompanied by cognitive decline (Michalik et al., 2004; Nardacchione et al., 1999; van de Warrenburg et al., 2001). The ataxin-7 gene encodes a protein which is now known to be a component of the STAGA transcription co-activator complex, which plays a critical role in regulating gene expression (Helmlinger et al., 2004; Palhan et al., 2005). STAGA is involved in the transcriptional control of genes crucial for neuronal function, survival, and DNA damage repair (Martinez et al., 2001). Despite our realization that ataxin-7 is a core component of the STAGA complex, the underlying pathological mechanisms leading to the specific signature of SCA7 disease remain elusive.

Studies in model mice have underscored the importance of ataxin-7 in neural function, as expression of polyQ-expanded ataxin-7 yields phenotypes representative of SCA7 neurodegeneration, including cone-rod dystrophy retinal degeneration, cerebellar degeneration, brainstem pathology, and cortical dysfunction (La Spada et al., 2001; Yoo et al., 2003; Yvert et al., 2001). Ataxin-7 is ubiquitously expressed, and ataxin-7 localizes extensively to both the nucleus and the cytosol. This subcellular localization pattern is facilitated by the presence of three nuclear localization signals (NLS) and one nuclear export signal (NES) in the ataxin-7 protein, allowing it to actively shuttle between the cytosol and the nucleus through nuclear pore complexes (NPCs) via nucleocytoplasmic (N/C) transport (David et al., 1997; Holmberg et al., 1998; Kaytor et al., 1999; Mauger et al., 1999; Trottier et al., 1995).

N/C transport involves both passive and active mechanisms. Smaller molecules (<40 kDa) can passively move, while larger macromolecules require active transport. The active N/C transport process relies on NPCs, composed of ~30 distinct proteins called nucleoporins (NUPS), which form long-lived aqueous protein channels (Zeitler and Weis, 2004). Apart from NPCs, karyopherins also play crucial roles in facilitating nuclear import and export. Importin-α and importin-β function in the cytosol by binding to a NLS to facilitate nuclear import, while exportins enable nuclear export by binding to a NES. The release of cargo from karyopherins is triggered by RanGap1 through RanGTP hydrolysis (Fahrenkrog and Aebi, 2003; Newmeyer, 1993). The cleavage of mutant ataxin-7 by caspase-7 leads to the formation of a truncated ataxin-7 peptide that lacks its nuclear NES (Young et al., 2007). This process promotes the development of nuclear inclusions (NIs) in cells, consisting of short fragments of mutated misfolded ataxin-7 protein, along with various other proteins. While NIs are believed to be involved in the degenerative process, it is noteworthy that unaffected cells also contain NIs, suggesting that large NIs alone do not solely account for neuronal demise (David et al., 1998; Holmberg et al., 1998).

N/C transport is often altered in the pathogenesis of neurodegenerative proteinopathies, including especially amyotrophic lateral sclerosis (ALS), Huntington’s disease (HD), and Alzheimer’s disease (Li and Lagier-Tourenne, 2018). For example, previous studies have suggested a link between neurodegenerative disease pathology and nuclear membrane abnormalities, such as invaginations and protrusions (Gasset-Rosa et al., 2017; Giampetruzzi et al., 2019; Paonessa et al., 2019). In HD mice and human cortical tissue, immunostaining analysis has demonstrated marked alterations in nuclear envelope morphology (Gasset-Rosa et al., 2017). Similarly, ultrastructural analysis of Neuro2a cells transfected with mutant profilin-1, which can cause ALS, revealed severe defects in the structure of the nucleus, with frequent folds, invaginations, and protrusions that are not observed in cells expressing normal profilin-1 (Giampetruzzi et al., 2019). Immunostaining of iPSC-derived cortical neurons from frontotemporal dementia (FTD) patients with autosomal dominant mutations in the *MAPT* gene, encoding tau protein, uncovered marked differences in nuclear morphology between non-demented controls and tau mutant neurons, with the presence of large folds or invaginations of the Lamin B1-positive inner nuclear lamina (Paonessa et al., 2019). To better delineate the molecular mechanisms responsible for SCA7 disease pathogenesis, we chose to examine the role of N/C transport in SCA7 cellular pathology. Although we did not detect any nuclear morphology abnormalities or N/C transport defects in SCA7 model mice, we did uncover evidence for increased nuclear import in neuronal cells derived from SCA7 patient stem cells. Our findings offer unexpected insights into the intricate pathophysiology of SCA7 and suggest that not all protein aggregation disorders affect N/C transport function in the same way.

## 2 Methods

### 2.1 Mouse studies

The derivation and characterization of the SCA7 266Q knock-in mice has been described (Yoo et al., 2003). SCA7-266Q mice (B6.129S7-Atxn7^tm1Hzo^/J; JAX#:008682) were maintained on a C57BL/6J background at the Duke University DLAR facility and UC Irvine ULAR facility. For all experiments, female and male mice were equally represented to control for sex differences. 8.5-week-old symptomatic 266Q mice and WT littermates were transcardially perfused with 20 mL of 1X PBS, then 20 mL of 4% paraformaldehyde (PFA). Following the perfusion, the intact brain and eyes were dissected out. While the brain was immediately equilibrated in 30% sucrose/PBS, the eyes were snipped at the corneas and placed in 4% PFA overnight and then placed in 30% sucrose. After 48–72 h in sucrose, both murine tissues were then embedded in OCT (Tissue-Tek). Twenty micrometer coronal sections, for both tissues, were collected on a Leica CM3050s cryostat directly onto slides.

For immunohistochemistry, sections were blocked in 5% goat serum in 1X PBS and 0.1% Triton X-100 for 1 h at room temperature. For ataxin-7 aggregate staining and to assess nuclear membrane morphology, sections were incubated for 24 h in primary antibodies: Mouse anti-RanGap1 (1:250, Cat. No. sc-28322, Santa Cruz) and Rabbit anti-Ataxin-7 (1:500, Cat. No. PA1-749, Thermo). To control for antibody variability, a follow up stain for nuclear membrane abnormalities in cerebellar tissue was done using Rabbit anti-Lamin B1 (1:250, Cat. No. ab16048, Abcam) and Mouse anti-Calbindin (1:500, Cat. No. C9848-100UL, Sigma). To quantify nuclear membrane abnormalities in retinal neurons, retinal sections were incubated in Anti-Rhodopsin (1:500, Cat. No. ab5417, Abcam) and Rabbit anti Lamin-B1. Secondary antibodies used were as follows: goat anti-mouse Alexa Fluor PLUS 594 (1:500, Cat. No. A-11032, Thermo), goat anti-rabbit Alexa Flour 488 (1:500, Cat. No. A-11034), goat anti-rabbit Alexa Fluor PLUS 555 (1:500, Cat. No. A32732) and goat anti-mouse Alexa Fluor PLUS 488 (1:500, Cat. No. A32723). Following the secondary antibody incubation, all sections were incubated in Hoechst 33342 (1:10000, Cat. No. H1399, Thermo) for 5 min to identify nuclei. Sections were imaged using a Nikon A1 confocal at 10×, 20×, and 60× magnification. Experimenter was blind to genotype during imaging and analysis.

### 2.2 Neuron progenitor cell differentiation and immunostaining analysis

The generation and characterization of iPSC’s from SCA7 patients and first-degree unaffected relatives has been described (Ward et al., 2019). iPSCs were grown and expanded on Matrgiel-coated 60 mm plates using StemFlex medium (Invitrogen) and were passaged using ReleSR (StemCell Technologies). After 3 passages, iPSCs were split into Matrigel-coated 24-well plates at a cell density of ~0.5 E6 cells/mL to begin neural induction. Differentiation of iPSCs into neuron progenitor cells (NPCs) was performed using PSC Neural Induction Medium (Invitrogen) and the published PSC NIM protocol (#MAN0008031). After 7 days, newly differentiated NPCs were passaged with Accutase and re-plated into Matrigel-coated 6-well plates at ~1.0 × 10^6^ cells/well. NPCs were passaged using Neural Induction Medium (NIM, 50:50 Neurobasal: Advanced DMEM/F12 and 1× neural induction supplement). NPCs were passaged in NIM medium for 3 passages before cryopreservation at P3. After 3 passages, NPCs medium was changed to Neural Maintenance Medium (50:50 Neurobasal: Advanced DMEM/F12, 5 μg/mL) Heparin (Sigma), 20 ng/mL FGF2 (Thermo Fisher) and 20 ng/mL EGF (Thermo Fisher). NPCs were identified by immunofluorescence and antibodies against Nestin (Santa Cruz, 1:200, Ms) and Pax6 (Covance, 1:200, Rb).

NPCs generated from SCA7 patient derived iPSCs and first-degree unaffected relatives were incubated in 4% PFA for 15 min. Following the PFA incubation, cells were rinsed thoroughly in 1X PBS and blocked in 5% goat serum in 1X PBS and 0.1% Triton X-100 for 1 h at room temperature. To stain for Pax6 and Nestin, cells were incubated for 24 h in Rabbit anti-Pax6 (1:500, Cat. No. 42-6600) and Mouse anti-Nestin (1:500, Cat. No. sc-23927) respectively. Secondary antibodies used were goat anti-rabbit Alexa Fluor PLUS 555 (1:500, Cat. No. A32732) and goat anti-mouse Alexa Fluor PLUS 488 (1:500, Cat. No. A32723). Following the secondary antibody incubation, cells were incubated in Hoechst 33342 (1:10000, Cat. No. H1399, Thermo) for 5 min to identify nuclei. Sections were imaged using a Nikon A1 confocal at 20× magnification.

### 2.3 Cortical neuron differentiation and immunostaining analysis

Using dual SMAD inhibition, iPSCs were differentiated to cortical neurons following the Young lab’s protocol described here (Mishra et al., 2022). Cell lines were maintained at 37°C in a 5% CO_2_ incubator. Following the differentiation protocol, immunocytochemistry was performed to confirm culture was highly enriched for cortical neurons.

Cortical neurons generated from SCA7 patient derived iPSCs and first-degree unaffected relatives were incubated in 4% PFA for 15 min. Following the PFA incubation, cells were rinsed thoroughly in 1X PBS and blocked in 5% goat serum in 1X PBS and 0.1% Triton X-100 for 1 h at room temperature. To stain for mature neuronal markers TBR1 and MAP2, cells were incubated for 24 h in rabbit anti-TBR1 (1:500, Cat. No. ab183032, Abcam) and chicken anti-MAP2 (1:500, Cat. No. ab92434, Abcam), respectively. Secondary antibodies used were goat anti-rabbit Alexa Fluor PLUS 488 (1:500, Cat. No. A32731) and goat anti-chicken Alexa Fluor PLUS 555 (1:500, Cat. No. A32732). Following the secondary antibody incubation, cells were incubated in Hoechst 33342 (1:10000, Cat. No. H1399, Thermo) for 5 min to identify nuclei. Sections were imaged using a Nikon A1 confocal at 40× magnification and Echo Revolve at 20× magnification.

### 2.4 FRAP experiments

Four different NPC lines from SCA7 patients and first-degree unaffected relatives were used for FRAP experiments. One batch of experiments were carried out with stem cells from a 10Q/10Q unaffected father and his SCA7-affected 65Q/10Q daughter. The other batch of live cell experiments used stem cells from an unaffected 10Q/10Q mother and her SCA7-affected 70Q/10Q son. Three independent trials of FRAP for each of these cell lines were conducted. First, the 70Q/10Q cells and their 10Q/10Q control cell line was transduced with shuttle NLS-NES-eGFP lentivirus. This virus contained a GFP reporter carrying an NLS and NES signal that was generated by inserting a fusion of EGFP (Clontech) and the NLS/NES together with an HA-tag into Kpn1 and XbaI sites of pcDNA3.1 myc-His A (Woerner et al., 2016). Using a standard protocol, the NLS-NES-eGFP was then triple transfected with pMD2.G and R8.74 into HEK293T cells to produce lentivirus particles. Following the 18-h transduction, the virus was removed from the cells and they were given 72 h to maximize GFP expression. Given that most of the GFP expression was found restricted to the cytoplasm in our NPCs, export blocker Leptomycin B (LMB, Sigma) was used to promote accumulation of nucleoplasmic GFP. Prior to live cell imaging, transduced NPCs were treated with 5 ng/mL of LMB for 15 min. Immediately following the 15 min, cell nuclei were photobleached and the nuclear florescence recovery per minute was recorded over the course of 10 min. Second the 65Q/10Q and their 10Q/10Q control cell lines were transduced with a CMV-NLS-td-Tomato-NES construct: NLS-tdTomato-NES (Grima et al., 2017). Due to appropriate levels of td-Tomato nuclear expression, no LMB was required for these FRAP experiments. As with the first set of NPC FRAP experiments, cell nuclei were photobleached and the nuclear florescence recovery per minute was recorded over the course of 10 min. FRAP data was then exported to excel for analysis.

Cortical neurons were generated from four different patient iPSC lines. One batch of experiments were carried out with neurons generated from a 10Q/10Q father and his 65Q/10Q daughter. The other batch of neurons were generated from a 10Q/10Q mother and her 70Q/10Q son. As with the NPC FRAP experiments, we conducted three independent trials of FRAP for each of these cell lines. Differentiated cell lines were transduced with a CMV-NLS-td-Tomato-NES construct: NLS-tdTomato-NES (Grima et al., 2017). After implementing the same transduction timeline as with NPCs, cell nuclei were bleached and fluorescence recovery data was collected over the course of 10 min.

### 2.5 Immunoblotting analysis

NPC nuclei were isolated using a nuclei pure prep nuclei isolation kit (SIGMA, NUC-201). Protein samples from NPCs nuclei were prepared by adding protease and phosphatase inhibitors (Thermo Fisher, 87786) and sonicating 5 min at 4°C (Bioruptor Pico). Total protein was quantified with Qubit Protein Assay (Thermo Fisher, Q33211). Lysates were mixed with 4× Laemmli Sample Buffer (Bio-Rad, 1610747) and 2-mercaptoethanol, and then heated at 95°C for 5 min. Lysates were electrophoresed by SDS-PAGE using 4–20% Stain-Free TGX gels (Bio-Rad, 5678093) and transferred to 0.45 μm PVDF membrane (Bio-Rad, 1704157). Membrane was blocked in 5% non-fat dry milk in TBST (1X TBS with 0.05% Tween-20) for 30 min at RT, and incubated with POM121 (Novus Biologicals, NBP2-19890) antibody overnight at 4°C and with goat anti-rabbit HRP (Thermo Fisher, A16110) for 60 min at room temperature. Detection was performed using SuperSignal West Pico PLUS (ThermoFisher, 34580) and imaged on a ChemiDoc MP system (Bio-Rad). Densitometry analysis was performed using ImageJ and normalized to total protein as detected by stain-free technology (Bio-Rad).

### 2.6 Formulae and statistics

All data were analyzed using Microsoft excel & Prism. To quantify the contour ratio using Excel, we incorporated the area and perimeter measurements into the equation $\frac{4\pi \times \text{area}}{{\text{perimeter}}^{2}}$. The contour ratio is represented on a scale from 0 to 1, with 1 being the most circular and 0 being the least (Scharner et al., 2011). Nested *t*-test was used to evaluate the percent of cells with nuclear membrane convolutions and contour ratio results, and significance (*α*) was set at *p* < 0.05.

## 3 Results

### 3.1 Cortical, cerebellar, and retinal cells have normal nuclear membrane morphology

**Figure 1:**
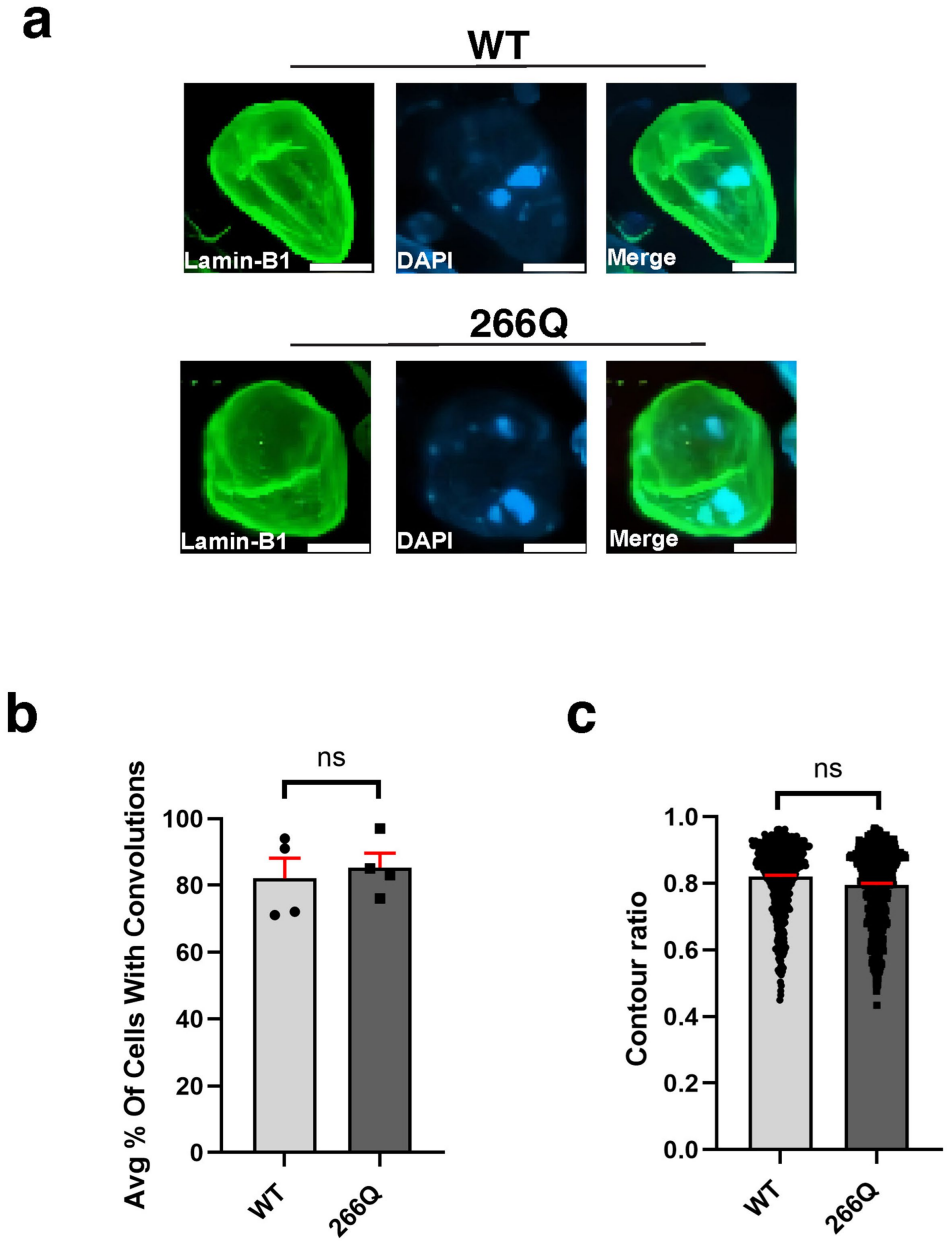
Purkinje cell neurons from SCA7 mice do not display nuclear membrane morphological defects. **(a)** Confocal microscopy images of mouse cerebellum from 8.5-week-old littermate control mice (WT) (*n* = 5) and from SCA7 266Q knock-in mice (SCA7) (*n* = 5) reveal no significant difference in the **(b)** % of Purkinje cells with convolutions, *p* = 0.6799 or in the **(c)** contour ratio, *p* = 0.6498. Lamin-B1 (green) was used to label the nuclear membrane. Data were collected from 100–150 cells per mouse. Data are represented by mean values ± SEM. *p* > 0.05 for nested *t*-test. White scale bars = 5 μm.

An emerging theme in the study of neurodegenerative proteinopathies is the presence of nuclear membrane pathology (Bennett et al., 2018), as documented for HD, a CAG-polyQ repeat expansion disorder (Grima et al., 2017). To determine if nuclear membrane pathology is a feature of SCA7, we evaluated nuclear membrane morphology in the SCA7 266Q knock-in mouse model (Yoo et al., 2003), selecting three brain regions that display progressive neurodegeneration: cerebellum, retina, and hippocampus. To assess nuclear membrane morphology, we performed immunohistochemistry (IHC) for nuclear membrane marker Lamin B1 and focused on Purkinje cell neurons in the cerebellum, because this cell type undergoes prominent degeneration. When we quantified Purkinje cell nuclear membrane morphology in symptomatic SCA7 266 mice and non-transgenic, age-matched wild-type (WT) littermate controls by counting the number of Purkinje cells, marked by calbindin, with nuclear membrane convolutions (Figure 1a), we did not observe a significant difference, as 86% of SCA7 266Q and 82% of WT Purkinje cells displayed such nuclear membrane convolutions (Figure 1b). In addition to counting cells with nuclear membrane convolutions, we determined nuclear circularity by conducting a contour ratio analysis, and we found that Purkinje cell nuclear contour ratios are similar in affected SCA7 266Q mice and WT littermate controls (Figure 1c). To confirm that SCA7 266Q mice are undergoing accumulation of misfolded polyQ-expanded ataxin-7 protein, we performed IHC on cerebellar sections from symptomatic SCA7 266Q mice and documented prominent aggregate pathology in cerebellar Purkinje cells (Supplementary Figure S1).

**Figure 2:**
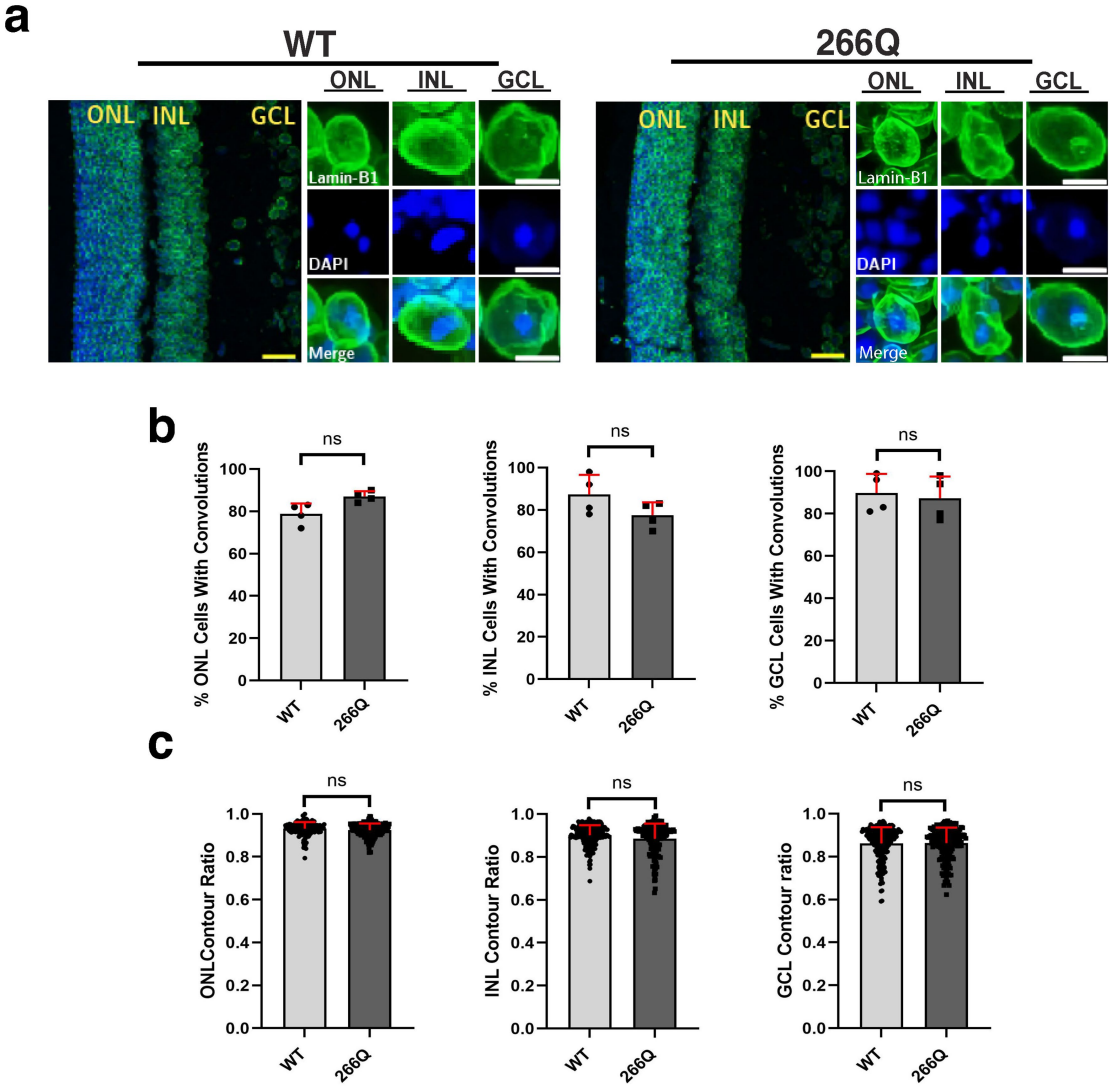
Immunostaining analysis reveals no significant differences in nuclear membrane morphology in the retina of SCA7 mice. **(a)** We conducted 2D analysis on confocal microscopy images of mouse retina from 8.5-week-old littermate control mice (WT) (*n* = 4) and from SCA7 266Q knock-in mice (SCA7) (*n* = 4) mice. **(b)** There were no significant differences in the percent of cells with convolutions from the outer nuclear layer (ONL), *p* = 0.0955; inner nuclear layer (INL), *p* = 0.2321; and the ganglion cell layer (GCL), *p* = 0.7282. **(c)** There were also no significant differences in the contour ratio from the ONL, *p* = 0.0791; INL, *p* = 0.5419; or the GCL, *p* = 0.9683. Lamin-B1 (green) was used to label the nuclear membrane. Data were collected from 100–150 cells per mouse. Data are represented by mean values SEM. Nested *t*-test used for statistical analysis. Yellow scale bars = 20 μm. White scale bars = 5 μm.

One unique clinical aspect of SCA7 is cone-rod dystrophy retinal degeneration characterized by photoreceptor degeneration and loss (La Spada et al., 2001). To determine if SCA7 model mice display nuclear membrane pathology while undergoing retinal degeneration, we performed IHC on retinal sections from symptomatic SCA7 266Q knock-in mice and WT littermate controls by immunostaining for Lamin B1 and counterstaining with DAPI (Figure 2a). When we quantified cells with nuclear membrane convolutions in the outer nuclear layer (photoreceptors), inner nuclear layer (bipolar cells; other neurons), and the ganglion cell layer (neurons that form the optic nerve), we found that SCA7 266Q knock-in mice and WT littermate controls display similar numbers of cells with nuclear convolutions on a percentage basis (Figure 2b), although we noted a trend toward increased numbers of outer nuclear layer cells with nuclear convolutions in SCA7 mice (*p* = 0.0955). We then calculated nuclear contour ratios, which corroborated the nuclear membrane convolution results, as SCA7 266Q mice and WT littermate controls exhibited similar nuclear contour ratios for outer nuclear layer, inner nuclear layer, and ganglion cell layer cells, though a trend toward decreased circularity was apparent for outer nuclear layer cells in SCA7 266Q mice (Figure 2c). We also confirmed the presence of intra-nuclear aggregates in each of these three cellular layers of the retina in symptomatic SCA7 266Q mice (Supplementary Figure S2).

**Figure 3:**
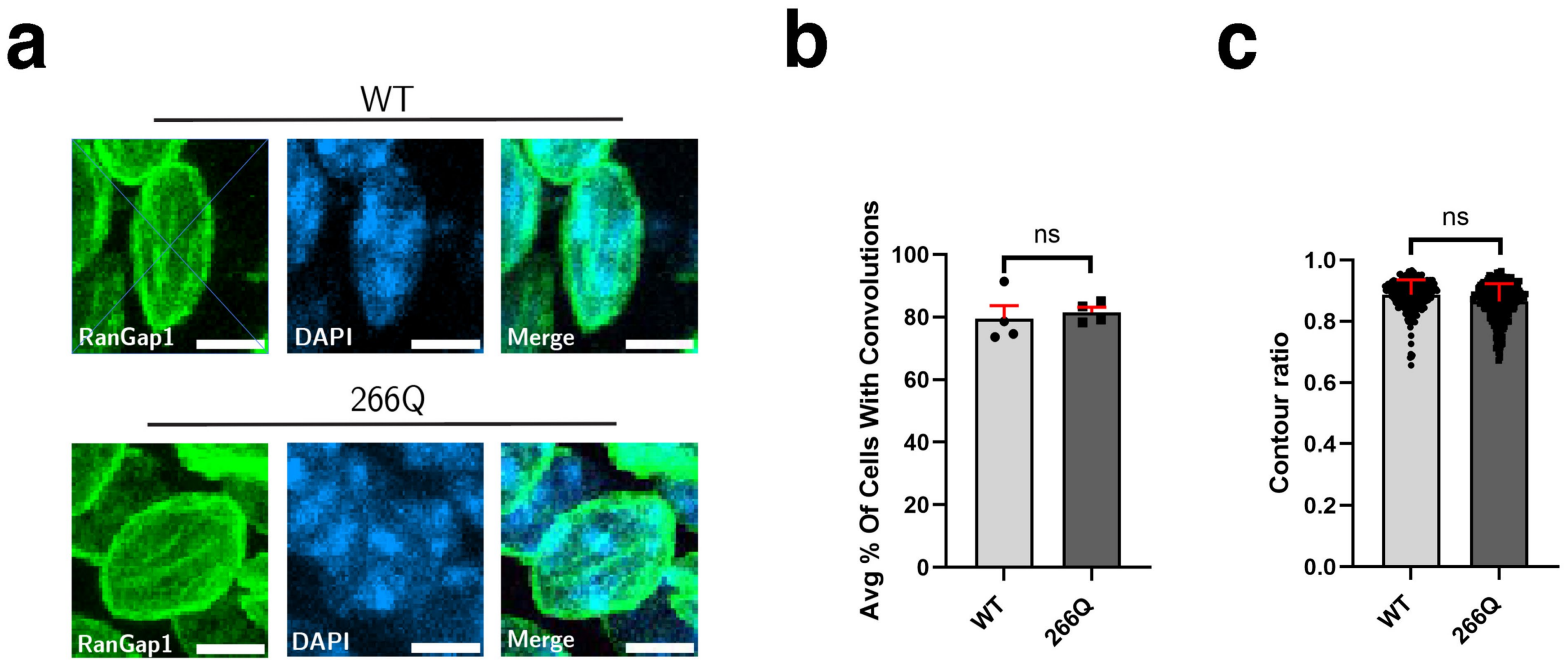
SCA7 mouse hippocampal tissue shows no significant difference in nuclear membrane morphology. **(a)** Conducting analysis on confocal microscopy images of mouse CA3 hippocampal neurons from 8.5-week-old littermate control mice (WT) (*n* = 4) and 266Q knock-in mice (SCA7) (*n* = 4) reveal no significant difference in the **(b)** percent of Purkinje cells with convolutions, *p* = 0.1306, or in the **(c)** contour ratio, *p* = 0.2611. RanGap1 (green) was used to label the nuclear membrane. Data were collected from 100–150 cells per mouse. Data are represented by mean values ± SEM. Nested *t*-test used for statistical analysis. White scale bars = 5 μm.

Although SCA7 predominantly involves the cerebellum, the brainstem, and the retina, a number of extra-cerebellar regions are pathologically affected, including the cortex and the hippocampus. To determine if symptomatic SCA7 mice display nuclear membrane pathology in the hippocampus, we immunostained sections of hippocampus from symptomatic SCA7 266Q mice and WT littermate controls for RanGap1 and counterstained with DAPI to examine nuclear membrane morphology (Figure 3a). We again observed no significant differences in the percentage of cells with nuclear membrane convolutions, as ~82% of SCA7 266Q hippocampal neurons showed nuclear membrane convolutions and ~80% of WT hippocampal neurons showed nuclear membrane convolutions (Figure 3b). Furthermore, analysis of nuclear contour ratios from SCA7 266Q mice and WT littermate controls indicated comparable circularity for hippocampal neuron nuclei (Figure 3c). To determine if aggregate accumulation occurs in the hippocampus of symptomatic SCA7 mice, we performed IHC with an anti-ataxin-7 antibody, and noted prominent aggregate formation in neurons in the dentate gyrus of the hippocampus (Supplementary Figure S3), confirming pathological involvement of this brain region in this SCA7 mouse model.

### 3.2 SCA7 mouse primary cortical neuron cultures have a normal nuclear import rate

**Figure 4:**
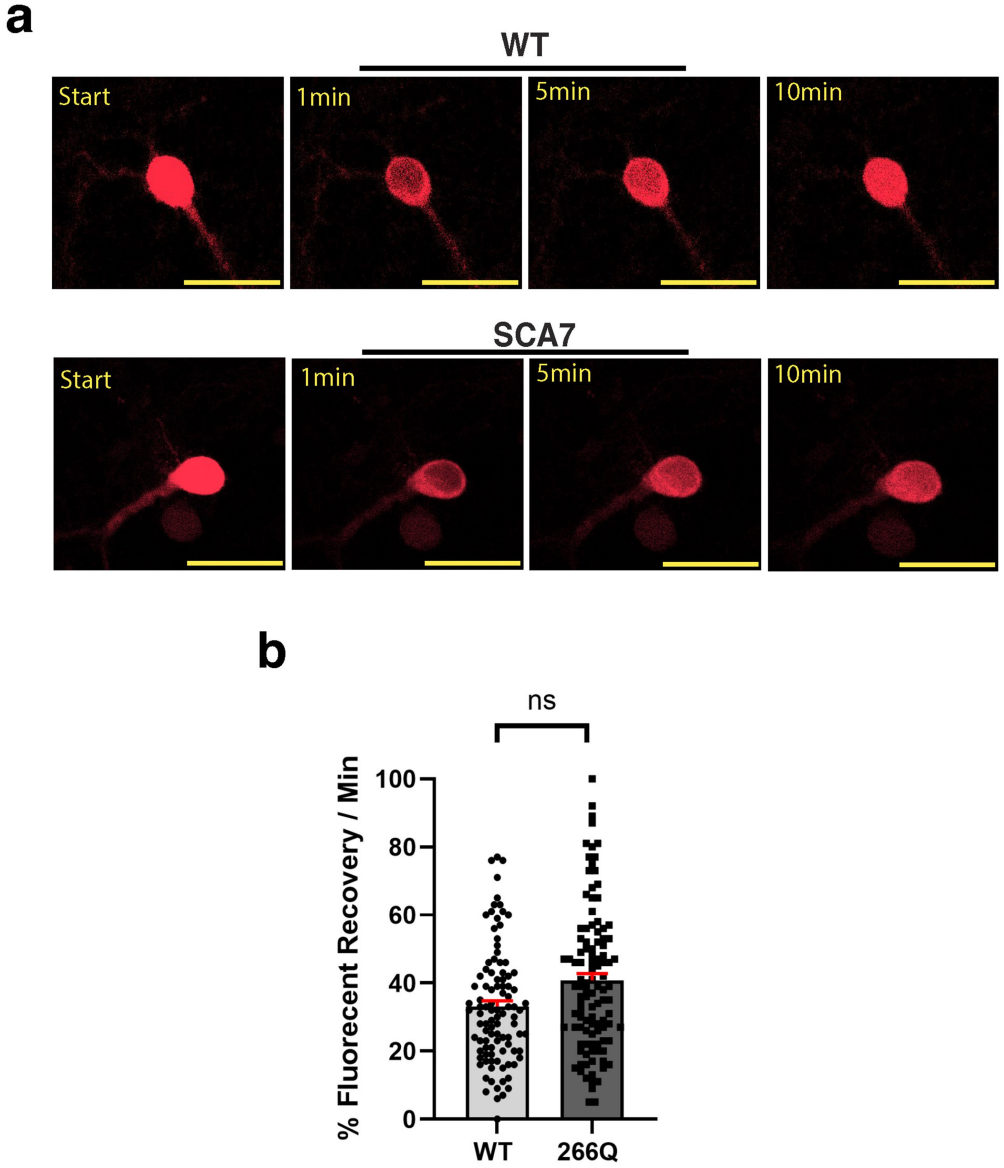
SCA7 mouse primary cortical neurons exhibit normal rates of nuclear import. **(a)** After photobleaching, the fluorescence recovery rate was collected over the course of 10 min. **(b)** The average percent of fluorescent recovery per minute for the NLS-tdTomato-NES lentivirus showed no significant difference in the nuclear import rate from 266Q SCA7 mouse primary cortical neurons compared to WT littermates, *p* = 0.3714. Fluorescent recovery was set to 100% for the highest value. Data were analyzed by nested *t*-test, and are represented by mean values ± SEM. Yellow scale bars = 20 μm.

Another read-out of nuclear membrane integrity and function is nucleocytoplasmic transport. To determine if nucleocytoplasmic shuttling function is affected in SCA7 266Q mice, we obtained a s-td-Tomato expression construct that contains both a NLS and NES (Eftekharzadeh et al., 2018), and packaged this s-td-Tomato shuttling vector reporter into lentivirus. We then cultured primary cortical neurons from neonatal SCA7 266Q mice and WT control littermates, and found that upon cortical neuron transduction with NLS-tdTomato-NES lentivirus, s-td-Tomato fluorescence was readily detectable in the nucleus (Figure 4a). To evaluate nucleocytoplasmic shuttling function in transduced neurons, we performed fluorescence recovery after photobleaching (FRAP) by selectively photobleaching the nucleus, and then measuring the rate of fluorescence recovery in the nucleus over 10 min (Figure 4a). There was no significant difference in the nuclear import rate of td-Tomato into primary cortical neurons from 266Q mice compared to WT mice (Figure 4b). Hence, nuclear membrane integrity and function appear intact in the central nervous system of SCA7 model mice.

### 3.3 Neurons derived from SCA7 patient iPSCs display increased nuclear import

**Figure 5:**
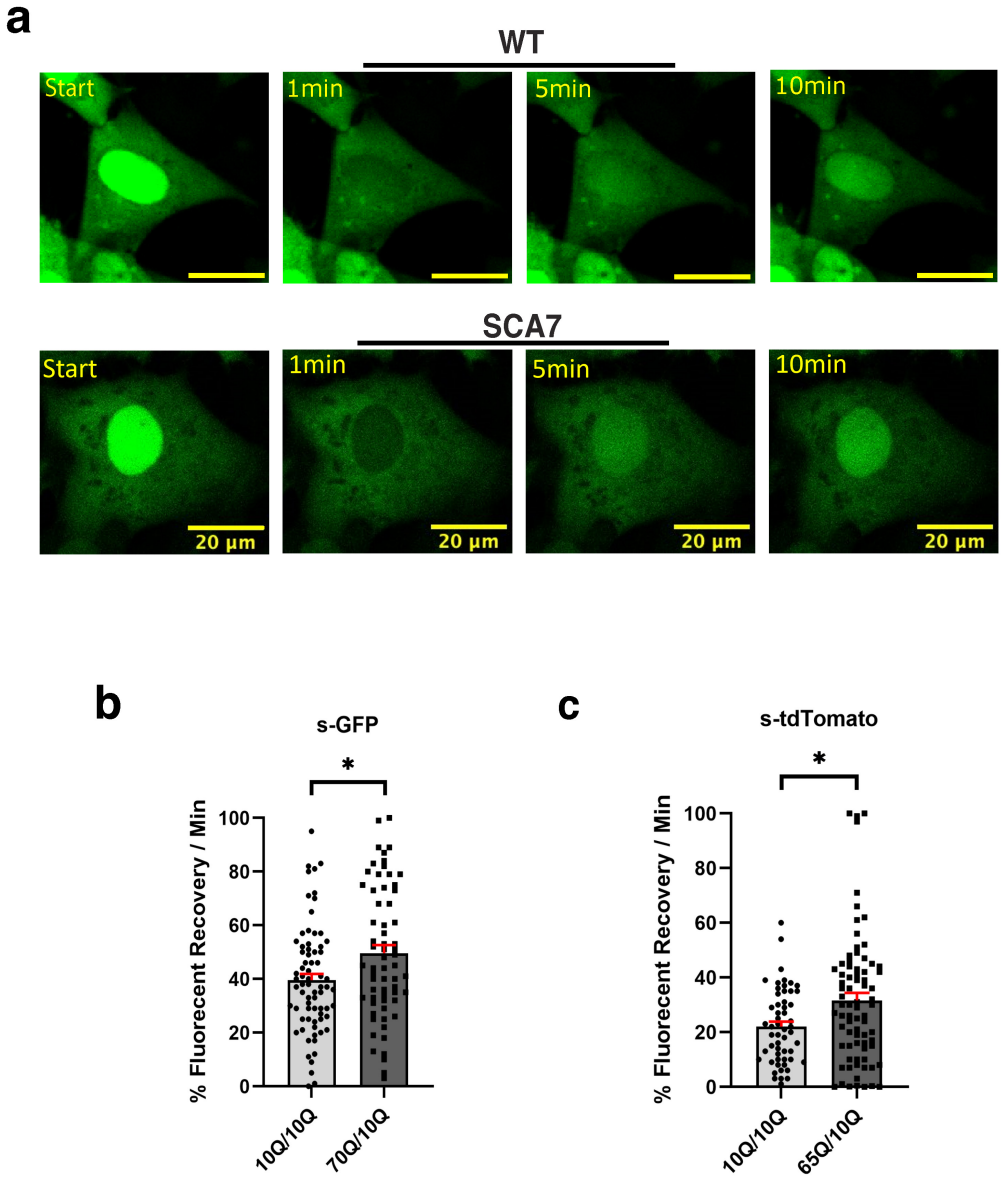
SCA7 NPCs display increased rates of nuclear import. **(a)** After photobleaching, the fluorescence recovery rate was collected over the course of 10 min. **(b,c)** The average percent of fluorescent recovery per minute for NLS-NES-s-GFP transduced 70Q/10Q DIV16 NPCs, and NLS-tdTomato-NES transduced 65Q/10Q DIV16 NPCs was significantly higher than for either of their respective control 10Q/10Q NPC lines: *p* = 0.0194 and *p* = 0.0321, respectively. Data were collected from 18 cells per line; *n* = 3 biological replicates, *n* = 3 technical replicates. Fluorescence recovery was set to 100% for the highest value. Data were analyzed by nested *t*-test, and are represented by mean values ± SEM. Yellow scale bars = 20 μm.

**Figure 6:**
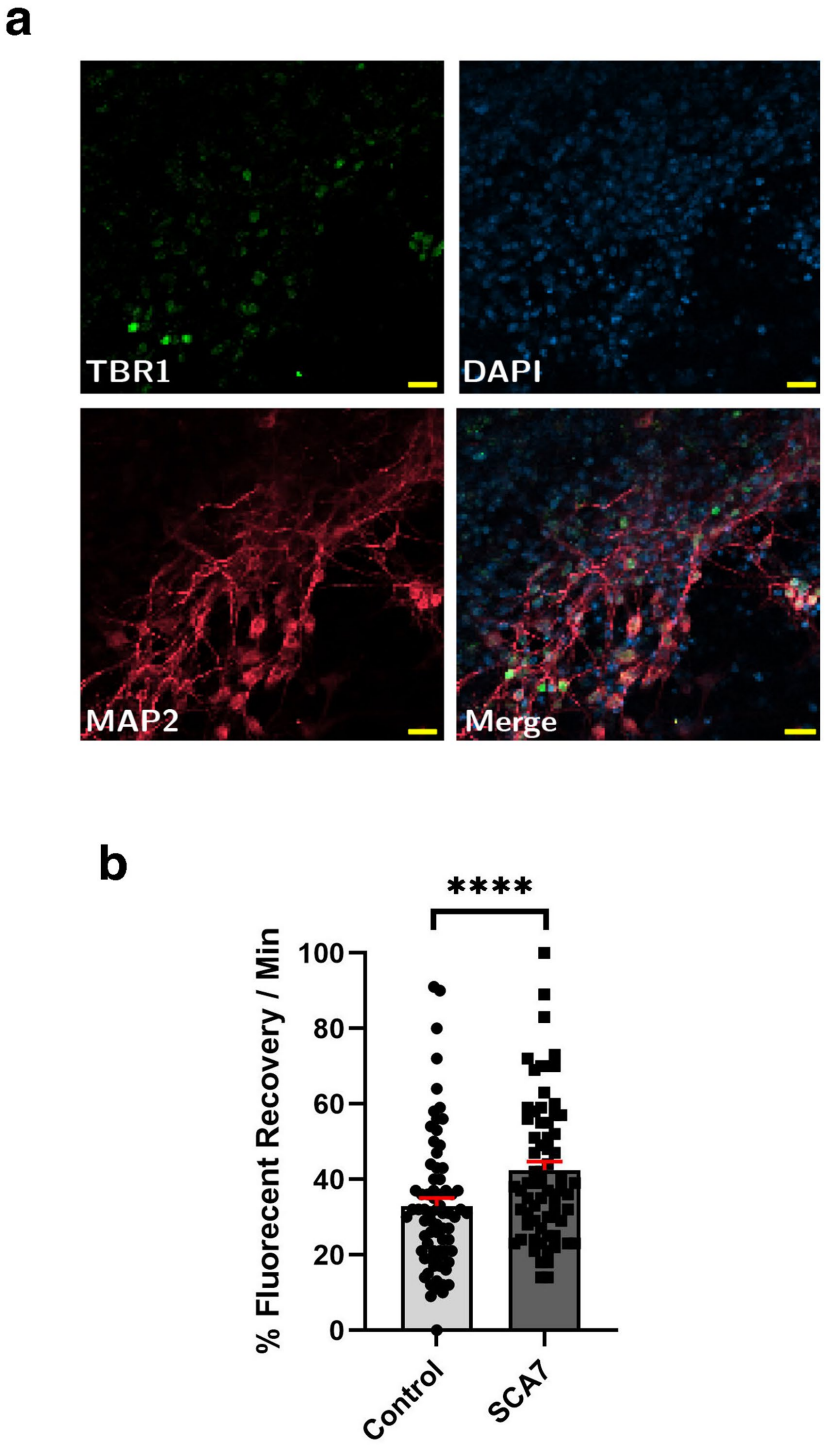
SCA7 patient iPSC-derived cortical neurons exhibit increased rates of nuclear import. **(a)** Neuronal markers TBR1 (green) and MAP2 (red) were used to label mature iPSC-derived cortical neurons. **(b)** After transducing neurons with the NLS-tdTomato-NES expression construct, we monitored fluorescence recovery rate over the course of 10 min. **(b)** Average percent of fluorescent recovery per minute for the SCA7 DIV50 iPSC-derived cortical neurons was significantly lower than for the control 10Q/10Q lines, *p* < 0.0001. Data were collected from 18 cells per line, and are represented by mean values ± SEM; *n* = 3 biological replicates, *n* = 4 technical replicates. Nested *t*-test was used for statistical analysis. Yellow scale bars = 20 μm.

Although SCA7 266Q mice are an excellent model of the disease process and recapitulate key features of the cognate human disease (Yoo et al., 2003), it is possible that certain aspects of the SCA7 disease process are not accurately represented. For this reason, we derived neuron progenitor cells (NPCs) from sets of induced pluripotent stem cells (iPSCs) generated from affected SCA7 patients and their unaffected first-degree relatives. Using either a NLS-tdTomato-NES lentivirus vector or a NLS-NES-eGFP lentivirus vector as done previously (Bennett et al., 2018), we transduced SCA7 and control NPCs with NLS-NES-eGFP lentivirus, and we performed fluorescence recovery after photobleaching (FRAP) by selectively photobleaching the nucleus and measuring the rate of fluorescence recovery in the nucleus over 10 min (Figure 5a). We observed markedly increased nuclear import in a SCA7 patient with 65 CAGs repeat and in an unrelated SCA7 patient with 70 CAG repeats, each, respectively, in comparison to their unaffected first-degree relative (Figures 5b,c). This unexpected finding prompted us to evaluate nuclear import in iPSC-derived cortical neurons by transducing SCA7 and control cortical neurons with the NLS-tdTomato-NES lentivirus and performing FRAP (Figure 6a). We observed significantly increased rates of nuclear import in SCA7 cortical neurons in comparison to unaffected first-degree relatives (Figure 6b), indicating that SCA7 neurons do display altered nucleocytoplasmic transport, but paradoxically this alteration is due to increased nuclear import.

### 3.4 Nuclear export is modestly, but not significantly decreased in SCA7 cortical neurons

**Figure 7:**
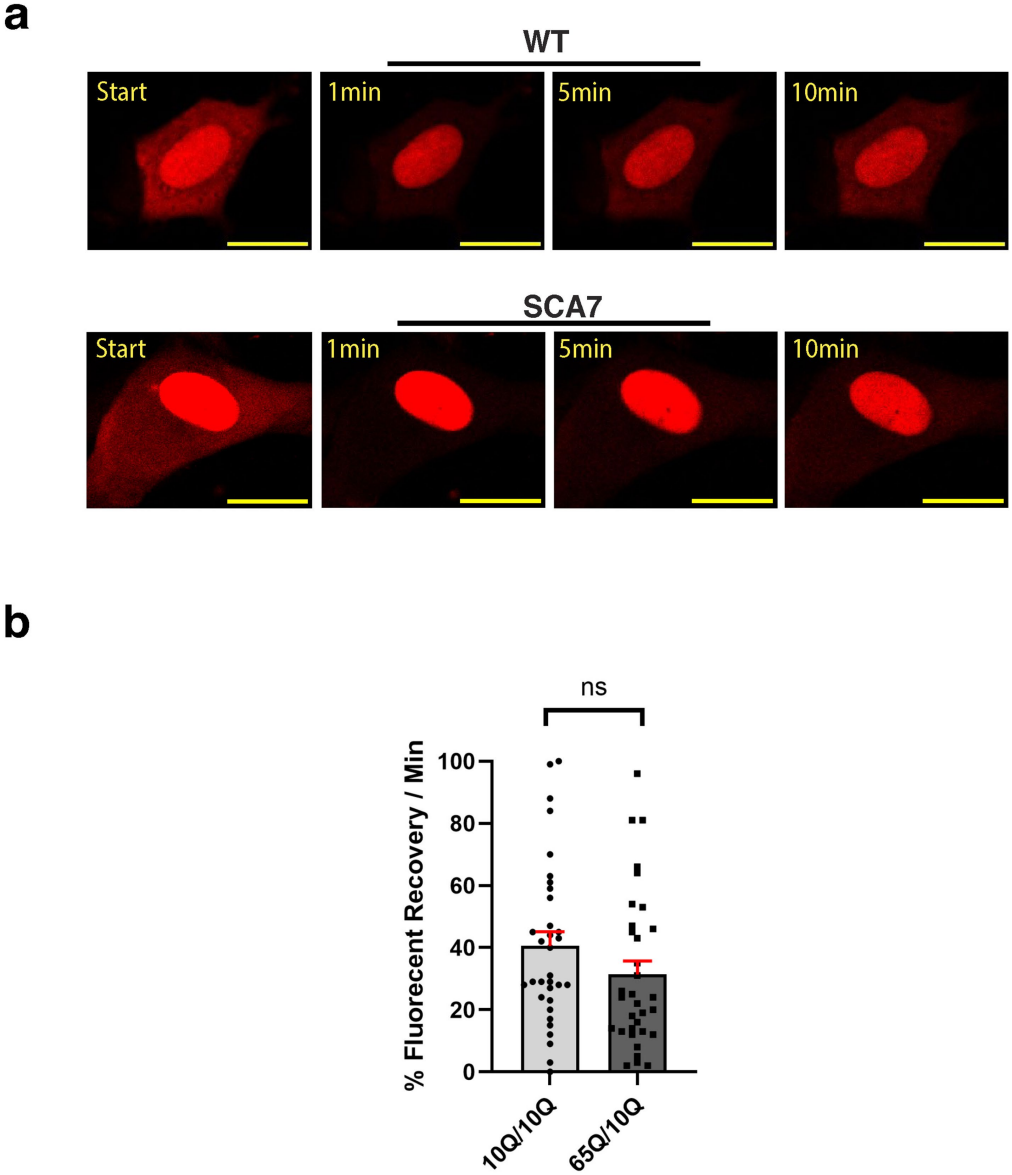
SCA7 patient neuron progenitor cells display a modest, but non-significant decrease in nucleocytoplasmic export. **(a)** We transduced neuron progenitor cells (NPCs) with the NLS-tdTomato-NES expression construct, and after photobleaching the surrounding cytosol, we monitored fluorescence recovery rate over the course of 10 min. **(b)** The average percent of fluorescent recovery per minute for the SCA7 65Q/10Q NPCs was modestly decreased with a non-significant trend in comparison to control 10Q/10Q NPCs; *p* = 0.2440. Data were collected from 12 cells per line, and are represented by mean values ± SEM, *n* = 3 biological replicates. Fluorescent recovery was set to 100% for the highest value. Data was then normalized to control mean and analyzed by nested *t*-test.

Because increased nuclear import could also reflect decreased nuclear export during the dynamic nucleocytoplasmic shuttling process, we set out to evaluate nuclear export in cortical neurons derived from SCA7 patients and unaffected first-degree relative controls. To do this, we again transduced cortical neurons with the NLS-tdTomato-NES lentivirus and performed FRAP, but this time we photobleached the surrounding cytosol and then tracked fluorescence recovery over 10 min (Figure 7a). We noted that the fluorescence recovery rate for the cytosol of SCA7 cortical neurons was 31% per minute, which was less than the fluorescence recovery rate of 41% per minute for control unaffected relative cortical neurons (Figure 7b). However, this difference only constituted a modest trend and was not significant (*p* = 0.244). Hence, the abnormally increased nuclear import observed in SCA7 neurons cannot be fully explained by slower nuclear export.

### 3.5 Levels of nucleoporin POM121 are reduced in SCA7 patient neurons

**Figure 8:**
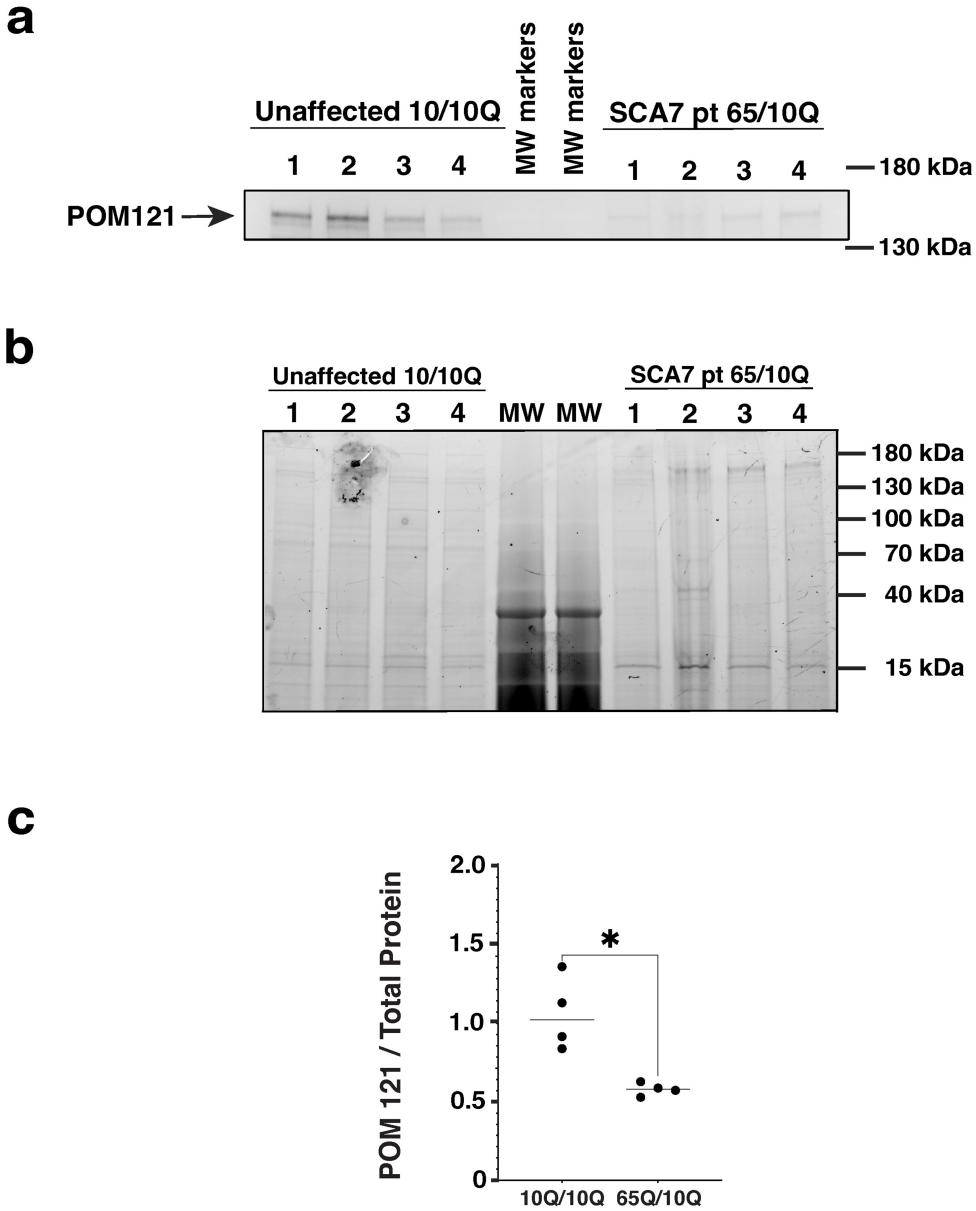
SCA7 patient NPCs display a marked reduction in the expression level of nucleoporin POM121. **(a)** We performed immunoblot analysis on neuron progenitor cells (NPCs) derived from either an unaffected related control (10Q/10Q) and on NPCs derived from a SCA7 patient (65Q/10Q) after obtaining protein lysates, and preparing and loading samples with identical amounts of total protein. Levels of full-length POM121 protein, which migrates at ~145 kDa, are visibly reduced in four SCA7 NPC biological replicates compared to four NPC biological replicates from the unaffected related control. **(b)** To assure equivalent loading and protein level normalization, we used a 4–20% Stain-Free TGX gel (shown here). **(c)** After quantifying POM121 protein levels by densitometry and normalizing to total protein using stain-free technology (BioRad), we noted a significant >40% reduction in POM121 protein expression in SCA7 NPCs. Data was normalized to unaffected control mean, which was arbitrarily set to 1.0, and then compared by nested *t*-test, *p* = 0.0286.

A number of studies have documented altered nucleocytoplasmic transport in neurodegenerative diseases, accompanied by altered expression of nucleoporin (NUP) proteins that comprise the nuclear pore complex. As previous work has implicated NUP POM121 as a regulator of NPC assembly (Funakoshi et al., 2011), and the expanded G4C2 repeat in C9orf72 ALS was found to reduce POM121 levels resulting in reduced expression of multiple NUPs (Coyne et al., 2020), we evaluated POM121 in SCA7 NPCs by immunoblot analysis. We observed a marked reduction in the expression level of full-length POM121 in SCA7 NPCs in comparison to NPCs obtained from an unaffected related control (Figure 8). These findings further indicate that NPC dysfunction is a feature of the SCA7 disease process.

## 4 Discussion

Recent studies on ALS and CAG-polyQ repeat expansion neurodegenerative disorders suggest that dysfunction of the nuclear membrane and impaired N/C transport may play crucial roles in disease pathogenesis (Kim and Taylor, 2017). To determine if nuclear membrane abnormalities or defects in N/C transport are occurring in SCA7, we examined SCA7 model mice and SCA7 patient iPSC-derived neurons. To evaluate SCA7 266Q knock-in mice for nuclear membrane abnormalities, we performed RanGap1 staining to delineate the nuclear membrane and we reconfirmed the existence of intranuclear ataxin-7 protein aggregates. As expected, we found intranuclear protein aggregates containing polyQ-expanded ataxin-7 in neurons of the cortex, cerebellum, and retina of SCA7 266Q knock-in mice. We investigated nuclear membrane morphology by staining for nuclear membrane marker Lamin B1 and compared it between SCA7 266Q mice and WT control littermates. To objectively quantify nuclear membrane morphology, we employed two different metrics: (1) average percent of cells with convolutions, and (2) nuclear contour ratio. While the former is a standard read-out for nuclear membrane abnormalities in neurodegenerative diseases (Bennett et al., 2018), the latter is a metric of membrane elongation and lobulation that incorporates membrane perimeter and area to quantify membrane roundness (Scharner et al., 2011). Unexpectedly, we found no significant differences in nuclear membrane morphology between SCA7 mice and WT controls in the cortex, cerebellum, or retina using either metric. This result is not unprecedented, however, as prior studies have not consistently linked nuclear membrane convolutions with neurodegenerative disease. For example, in C9orf72 ALS patient iPSC-derived motor neurons, all motor neurons progressively accumulate nuclear envelope invaginations, suggesting that they represent functional structural elements independent of pathology, and there was no difference in the frequency of nuclear invaginations between C9orf72 ALS patient-derived motor neurons and control iPSC-derived motor neurons (Ortega et al., 2020). In another study, immunostaining for DAPI and Lamin B1 demonstrated that nuclear morphology, as measured by sphericity and nuclear volume, was comparable between C9orf72 ALS patient iPSC-derived cortical neurons and control iPSC-derived cortical neurons (Coyne and Rothstein, 2021). However, using immunohistochemistry, this study went on to document significantly more nuclear invaginations in postmortem cortical sections obtained from C9orf72 patients. Hence, for certain neurodegenerative disorders, altered nuclear membrane morphology is not always present in model systems. In the case of SCA7, future studies should focus on analyzing retina, cortex, and cerebellum tissue obtained post-mortem from affected patients and appropriately matched controls to determine if the absence of nuclear membrane abnormalities in SCA7 model mice accurately reflects the situation in the human disease.

After analyzing nuclear membrane morphology, we sought to evaluate N/C transport. The results of these experiments, which employed fluorescence recovery after photobleaching (FRAP), revealed that SCA7 patient NPCs have a significantly higher rate of nuclear import than NPCs from unaffected first-degree relatives. As NPCs are still early in the process of becoming full-fledged neurons, we then derived fully differentiated cortical neurons from iPSCs from these same SCA7 patients and unaffected related controls. We observed significantly increased nuclear import in SCA7 iPSC-derived cortical neurons. Importantly, our findings are based upon stem cells derived from two different unrelated SCA7 families and at two different stages of neuron development using two different virally transduced constructs to quantify N/C transport. To our knowledge, SCA7 is the first neurodegenerative disorder found to display an increase in nuclear transport. All previous studies of neurodegenerative diseases performed to date have documented a decrease in N/C transport, when altered N/C transport was detected (Coyne et al., 2020; Eftekharzadeh et al., 2018; Grima et al., 2017; Kinoshita et al., 2009; Schilling et al., 1999; Sheffield et al., 2006). In addition to studying N/C transport in SCA7 patient iPSC-derived neurons, we also evaluated primary cortical neurons obtained from P0/P1 SCA7 266Q knock-in mice and WT littermate controls, but we did not observe any differences in N/C transport in mouse primary neurons. As neonatal mice may not accurately model age-dependent alterations in cellular homeostasis occurring in human SCA7 patients, the absence of altered N/C transport in this model system is not entirely unexpected. Indeed, previous studies have repeatedly shown that nuclear membrane integrity and nuclear transport function can become impaired with advancing age (Martins et al., 2020; Pujol et al., 2002). Hence, relying on neurons from newborn mice may simply not be an appropriate system for evaluating nuclear morphology defects and N/C transport.

If our finding of increased nuclear import is correct, what could be the underlying mechanism for this abnormality? And why would SCA7 be different from all other neurodegenerative proteinopathies studied to date? One possibility that we considered was an alteration in nuclear export, such that a decrease in nuclear export could be contributing to our detection of increased nuclear import. To test this hypothesis, we conducted FRAP experiments to assess nuclear export, but we did not find significant differences in nuclear export between NPCs from SCA7 patients and first-degree unaffected relatives, although we did see a trend towards decreased nuclear export in SCA7. While this trend cannot account for the markedly increased nuclear import detected in SCA7 patients, our results suggest that reduced nuclear export could be partially contributing to the increased nuclear import. To further examine the state of the NPC in SCA7 patients, we performed immunoblot analysis for POM121 on nuclei isolated from NPCs derived from SCA7 patients and unaffected first-degree relatives. We documented significantly decreased expression of POM121 in SCA7 NPC nuclei, indicating that NPC defects are likely present in SCA7 neurons and could be contributing to the altered N/C transport. Structured illumination microscopy (SIM) on nuclei isolated from C9orf72 patient iPSC-derived neurons has shown that a reduction in POM121 results in decreased levels of seven other nuclear pore proteins, thereby affecting the localization of RanGTPase, which is a key regulator of nuclear export (Coyne et al., 2020; Kalita et al., 2022). POM121 expression reductions in SCA7 could similarly be altering the stability of related nuclear pore proteins and consequently affecting N/C transport in SCA7; hence, similar studies should be conducted on SCA7 patient iPSC neurons in the future. One unique aspect of ataxin-7 protein normal function is that ataxin-7 subcellular localization is very dynamic, as it is constantly shuttling between the nucleus and the cytosol. Indeed, we have previously documented that ataxin-7 undergoes nuclear export via the CRM-1/exportin pathway, and that polyQ-expanded ataxin-7 exhibits decreased nuclear export (Taylor et al., 2006). As polyQ-expanded ataxin-7 interaction with the CRM-1/exportin pathway is impaired, it is likely that other regulators of N/C transport are similarly being disrupted, and these alterations in N/C transport could be unique to SCA7, resulting in increased nuclear import. Delineating how ataxin-7 interacts with the N/C transport machinery could yield important insights both into the mechanistic basis for increased nuclear import in SCA7 and the normal regulation of N/C transport.

Accumulation of misfolded proteins is a hallmark of many neurodegenerative disorders, including SCA7. In SCA3, mutant ataxin-3 forms intranuclear inclusions and nuclear localization of ataxin-3 is required for the development of SCA3 symptoms *in vivo*. Notably, transgenic models of SCA3 confirm that nuclear retention of ataxin-3 exacerbates the disease phenotype, while directing ataxin-3 out of the nucleus mitigates these effects (Schmidt et al., 1998; Bichelmeier et al., 2007; Lee et al., 2020). Similarly, in SCA7, ataxin-7 with an expanded polyQ tract accumulates in the nucleus. Histological analysis of the nuclear matrix extracted from SCA7-transfected cells suggests that polyQ-expanded ataxin-7 triggers pathology by altering the nuclear structure associated with the matrix and/or interfering with nuclear function (Kaytor et al., 1999). These findings underscore the importance of altered nuclear import and retention mechanisms in polyglutamine SCAs, reinforcing that the nuclear compartment is a common site of proteotoxicity in these disorders. Similarly, during aging, cells accumulate damaged proteins that interfere with their normal function, including especially proteins that comprise the nuclear membrane and nuclear pore. The development of N/C transport defects in a wide range of age-related neurodegenerative proteinopathies underscore that neurodegeneration could be viewed as decompensated aging. Future studies should be done to directly assay nuclear membrane leakage in SCA7 using Dextran permeability assays as well as more sophisticated imaging approaches, such as SIM, as such studies could further inform our understanding of SCA7 nuclear membrane pathology. Our results here suggest that altered N/C transport could play a role in the pathogenesis of SCA7, although it appears that the nature of the N/C transport defect in SCA7 is distinctly different from other related disorders. Understanding why SCA7 appears to be the exception to the rule may provide unexpected insights into the pathobiology of nuclear membrane dysfunction and N/C transport dysregulation in neurodegenerative diseases and may help guide development of novel therapeutic interventions for these devastating disorders.

## Data Availability

The original contributions presented in the study are included in the article/Supplementary material, further inquiries can be directed to the corresponding author.
