# Supplementary figures and images for: Increased nuclear import characterizes aberrant nucleocytoplasmic transport in neurons from patients with spinocerebellar ataxia type 7
Source: Front Mol Neurosci. 2024 Nov 22;17:1478110. doi: 10.3389/fnmol.2024.1478110 (PMC11621108; doi:10.3389/fnmol.2024.1478110)

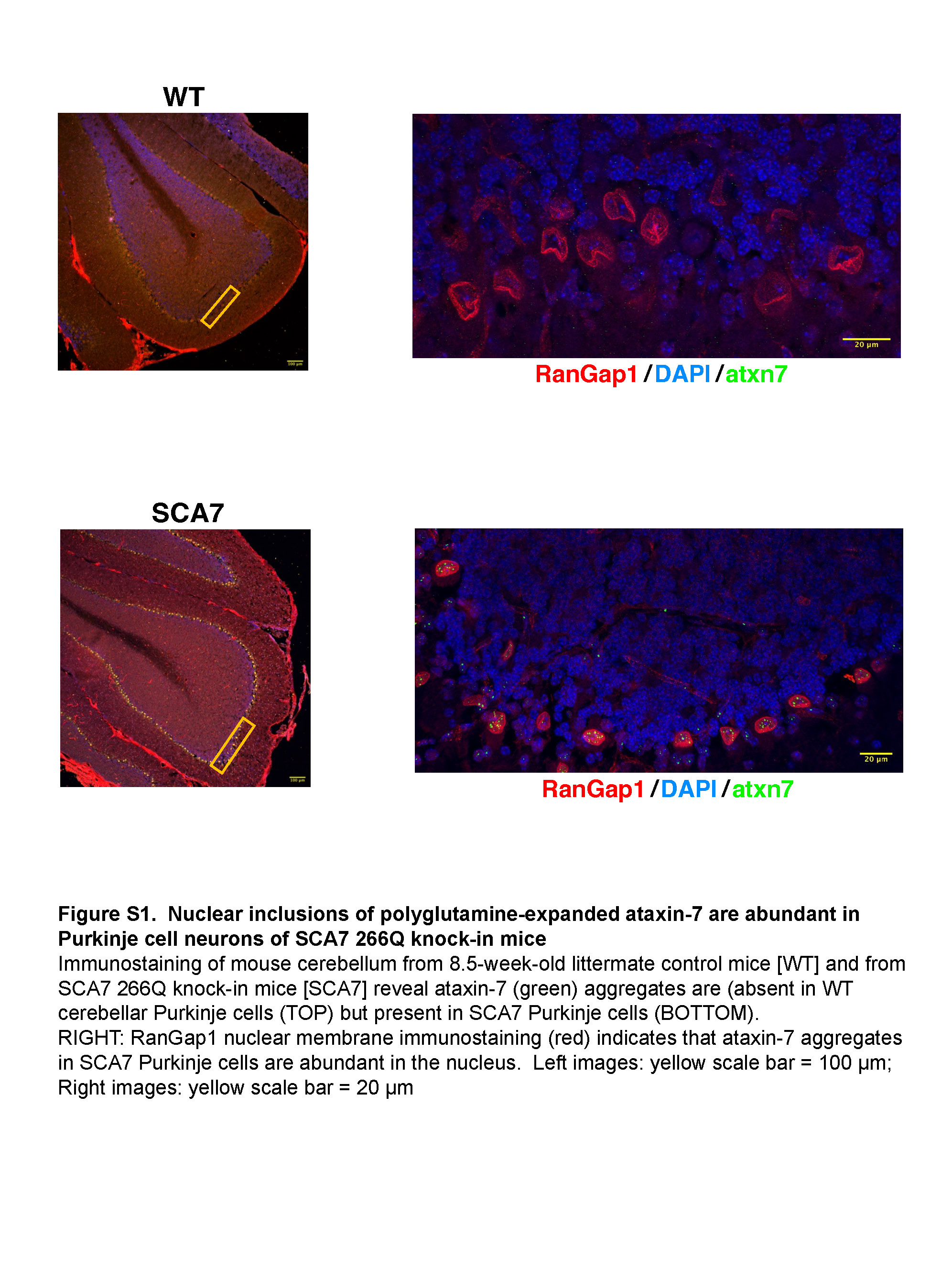

Supplement: Supplementary file 1 [file Image_1.jpeg]
